# Risk factors for the development of hospital-acquired pneumonia in patients with carbapenem-resistant *Acinetobacter baumannii* respiratory colonization and the role of multisite colonization: a multicenter retrospective study

**DOI:** 10.1007/s10096-025-05137-1

**Published:** 2025-04-28

**Authors:** Alessandro Russo, Francesca Serapide, Francesco Alessandri, Sara Palma Gullì, Andrea Bruni, Federico Longhini, Angela Quirino, Rocco Morena, Nadia Marascio, Giovanni Matera, Gabriella d’Ettorre, Veronica Zullino, Claudio Maria Mastroianni, Enrico Maria Trecarichi, Eugenio Garofalo, Giancarlo Ceccarelli

**Affiliations:** 1 Dipartimento di Scienze Mediche e Chirurgiche, Università “Magna Graecia”, Catanzaro, Italia; 2 Intensive Care Unit, Department of General and Specialistic Surgery, “Sapienza” University of Rome, Rome, Italy; 3 Intensive Care Unit, Department of Medical and Surgical Sciences, “Magna Graecia”, University of Catanzaro, Catanzaro, Italy; 4 Unit of Clinical Microbiology, Department of Health Sciences, “Magna Graecia” University of Catanzaro, Catanzaro, Italy; 5 Department of Public Health and Infectious Diseases, “Sapienza” University of Rome, Rome, Italy; 6 Infectious and Tropical Disease Unit, Department of Medical and Surgical Sciences, “Magna Graecia” University of Catanzaro, Viale Europa, 88100 Catanzaro, Italy

**Keywords:** *Acinetobacter baumannii*, Carbapenem-resistant, HAP, Multisite colonization, Ventilatory support

## Abstract

**Introduction:**

Carbapenem-resistant *Acinetobacter baumannii* (CRAB) infections are associated with high morbidity and mortality rates. Furthermore, the role of CRAB respiratory colonization, including multisite colonization, has not yet been adequately highlighted in critically ill patients.

**Materials and methods:**

In this retrospective multicenter study, conducted in 4 different Italian hospitals, patients with CRAB respiratory colonization +/- other site who developed or did not develop clinically significant pneumonia from December 2015 to December 2023 were enrolled. The primary objective of the study was to identify risk factors associated with the development of pneumonia.

**Results:**

760 patients were enrolled; among them, 392 (51.5%) developed pneumonia, while 304 (39.9%) patients presented multisite colonization. Overall, in-hospital mortality was 76.3% with a higher mortality (79.6%) in patients who developed pneumonia (*p* = 0.033). In logistic regression analysis, factors associated with the development of pneumonia included: age, immunosuppressive therapy, COPD, ventilatory support and multisite colonization. A score was developed with AUC 0.72, CI95% 0.68–0.75, *p* < 0.001 and a sensitivity of 79% and specificity of 55% with a score > 2 and a maximum score of 10 points. Multisite colonization was recorded more frequently in patients who developed pneumonia (51%, *p* < 0.001). Finally, Kaplan-Meier curves showed a significantly reduced survival at 30 days (*p* = 0.005) and throughout the hospital stay (*p* = 0.002) in patients with multisite colonization.

**Conclusions:**

This study highlights the risk factors associated with the development of pneumonia in patients already colonized by CRAB. Multisite colonization showed an important role as a risk factor for the development of pneumonia and for its correlation with mortality.

**Clinical trial number:**

Not applicable.

## Introduction

Carbapenem-resistant *Acinetobacter baumannii* (CRAB) is considered a global health emergency for nosocomial infections and epidemic outbreaks especially in critically ill patients in intensive care units (ICU) [1–4]. The rapid nosocomial spread of this infection is favored by the ability of CRAB to survive for long periods on surfaces, contaminating environments and promoting transmission to patients through healthcare personnel [2–6].

In over 50% of cases, the main manifestation of CRAB infection is hospital-acquired pneumonia (HAP), especially ventilator-associated pneumonia (VAP) [7]. For this reason, patients admitted to ICUs undergoing mechanical or non-invasive ventilator support and/or prolonged antibiotic treatment [8, 9, 11] are at higher risk of developing lower respiratory tract infection by CRAB [1, 4] with mortality rates ranging from 25 to 70% in different studies [1, 5, 6].

Of importance, most of these patients showed a CRAB colonization, especially of the respiratory tract, before developing a clinically significant infection [3, 7]. Distinguishing between colonization and HAP could be very difficult in ICU patients; to date, no clear risk factors have been identified for pneumonia development in colonized patients, and the role of multisite colonization by CRAB in increasing the risk of pneumonia or poor outcomes in ICU patients remains unclear in the literature.

The aim of this study was to evaluate risk factors for development of HAP in colonized patients admitted to ICU and to assess the role of multisite colonization by CRAB for the risk of pneumonia and mortality.

## Materials and methods

### Study design and patient selection

This was a multicenter, retrospective, cohort study including patients hospitalized in ICU from December 2015 to December 2023 in four Hospitals in Italy with a mean number of hospital and ICU beds of 750 and 25, respectively.

During the study period, active weekly surveillance was performed in these Hospitals (as reported in internal protocol for management of MDR infections). Cases were eligible for the study if the patient: (1) was aged ≥ 18 years, and (2) had a monomicrobial colonization by CRAB of the respiratory tract +/- other site. All patients admitted in ICU without evidence of CRAB colonization were excluded from the analysis. The study was conducted according to the principles stated in the Declaration of Helsinki and was approved by the local Ethics Committees (Regione Calabria, n° 19/2023). Collected data were anonymized and informed consent was waived.

Patient data were collected from medical charts and from computerized hospital databases or clinical charts using a pre-established questionnaire. The following data were reviewed: demographics; clinical and laboratory findings; comorbidities and Charlson Comorbidity Index; microbiological data; duration of ICU and hospital stay; other infections during ICU stay; treatment and procedures like non-invasive ventilation [NIV] and mechanical ventilation (defined as ventilatory support), continuous renal replacement therapy [CRRT], and/or extracorporeal membrane oxygenation [ECMO]; sequential organ failure assessment (SOFA) at time of infection; previous CRAB colonization; sites of CRAB colonization during ICU hospitalization; antibiotic therapy used before CRAB infection; antibiotic regimens used for CRAB infection; progression to septic shock; 30-day mortality.

### Microbiological identification and clinical definitions

The identification of CRAB strains was based on local laboratory techniques. From positive cultures, Gram staining and a rapid identification protocol were adopted. The bacterial pellet obtained directly from positive cultures was used for MALDI-TOF MS (Bruker Daltonics) identification and for molecular analysis. The Vitek 2 automated system (bioMérieux, Marcy l’Etoile, France) was used for isolate identification and antimicrobial susceptibility testing, except for colistin resistance, which was determined by broth dilution following EUCAST guidelines (The Vitek 2 automated system (bioMérieux, Marcy l’Etoile, France) was used for isolate identification and antimicrobial susceptibility testing. Minimum inhibitory concentrations (MICs) were established according to the European Committee on Antimicrobial Susceptibility Testing (EUCAST) breakpoints, including cefiderocol MIC values that were determined using Kirby-Bauer disc diffusion test on regular un-supplemented Mueller-Hinton (MH) agar (Liofilchem) and discs impregnated with 30 micrograms of drug (Liofilchem). A multiplex PCR (FilmArray, BioFire Diagnostics, BioMérieux, Salt Lake City, UT, USA) was used to identify the possible organisms in respiratory samples and blood cultures, when appropriate.

All CRAB isolates from the respiratory tract (throat swab, bronchoalveolar lavage, or bronchoaspiration), skin (axilla), or other sites (except urine) were considered primary colonization sites in the absence of clinical signs of infection. Isolation of CRAB from two or more different sites simultaneously was defined as multi-site colonization. The development of clinically significant pulmonary infections was classified according to the definitions established by the Centers for Disease Control and Prevention (CDC). Hospital-acquired pneumonia (HAP) was defined as pneumonia occurring at least 48 h after hospital admission. Ventilator-associated pneumonia (VAP) was defined as pneumonia occurring at least 48 h after the start of mechanical ventilation. Sepsis and septic shock were defined according to the Surviving Sepsis Campaign criteria.

### Endpoints and statistical analysis

The primary endpoint of the study was to evaluate risk factors associated with pneumonia development in the study population. Secondary endpoint was to evaluate in-hospital and 30-day mortality associated with multisite colonization.

Continuous variables were summarized as median and interquartile range (IQRs, 25-75%) or mean with standard deviation (± SD) according to normal distribution, and dichotomous variables as sample frequencies (n), proportions (or percentages) and rates of the given data on each variable. The normality of distributions was evaluated using the Kolmogorov-Smirnov test. To detect significant differences between groups, we used Chi-square tests or Fisher’s exact tests for categorical variables, and the 2-tailed Student’s t-test or Mann-Whitney U test for continuous variables, when appropriate. Statistical significance was assumed if the null hypothesis could be rejected at *p* < 0.05.

In a multivariate analysis of survival, the logistic regression model adjusted for confounding factors (including sex, age, Charlson Comorbidity Index, comorbidities including COPD and COVID-19, immunosuppressive therapy, SOFA score, septic shock, length of ICU and hospitaly stay, antimicrobial therapies) was tested using a proportional hazards model analysis with backward stepwise selection and *p* < 0.05 for all variables, to identify factors independently associated and determine the effects of all clinical and therapeutic variables on 30-day survival. Adjusted hazard ratios (HR) and 95% confidence intervals (CIs) were reported.

Kaplan-Meier curves were used to determine survival at 30 days and in-hospital in patients with multisite colonization. Survival curves for time-to-event variables, constructed using Kaplan–Meier estimates, were based on all available data and were compared using the log-rank test. Wald confidence intervals and tests for hazard ratio (HR) were computed based on the estimated standard errors. Possible confounding factors and interactions (as reported above) were weighted during analysis. Statistical significance was established at ≤ 0.05. All reported P-values are 2-tailed.

A predictive model of HAP development was constructed according to the ß-coefficients of each variable obtained from the multivariable analysis. We selected potentially useful baseline characteristic predictor variables by minimization of Akaike Information Criterion (AIC), given that the main target for analysis is prediction. For reasons of parsimony, we then discarded non-significant variables from the model minimizing AIC. The power of this model was assessed using the area under the receiver operating characteristic curve (AUROC). The performance of the derived model was also evaluated by use of the Hosmer-Lemeshow test (goodness-of-fit). Finally, internal validation was performed using bootstrapping techniques. We calculated sensitivity, specificity, negative and positive predictive values (with 95% confidence intervals) for the cut-off point of the score in order to predict the infection status. We also calculated negative and positive likelihood ratios (with 95% confidence intervals). The results obtained were analyzed using commercially available statistical software package (SPSS, version 29.0; SPSS Inc, Chicago, Illinois).

## Results

During the study period, a total of 760 patients were enrolled. Of these, 392 (51.5%) developed HAP, while 368 (48.5%) did not develop the infection. The mean time from ICU admission and CRAB colonization was 9.2 (± 6.7) days. Overall, 30-day mortality was 65.4% and in-hospital mortality 76.3%. Alla strains showed resistance to carbapenem with a rate of 12% and 8% of resistance to colistin and cefiderocol (when tested), respectively. Finally, CRAB colonization was assessed from throat swab in 73%, from bronchoalveolar lavage in 42%, from bronchoaspiration in 45%, from skin in 12%, and from rectal swab in 79% of patients.

As shown in Table 1, patients who developed HAP were significantly older than those without pneumonia (mean age 62.9 ± 15.7 vs. 59.2 ± 17.8 years; *p* = 0.015). The mean length of hospitalization was shorter in HAP patients (32.6 ± 22.2 vs. 43.5 ± 37.7 days; *p* < 0.001), as was the mean duration of ICU stay (25.6 ± 21.2 vs. 32.4 ± 32.2 days; *p* < 0.001). HAP patients also had a higher Charlson Comorbidity Index (8.33 ± 3.2 vs. 3.46 ± 3.9 points; *p* = 0.027), indicating greater baseline severity. Furthermore, the rate of septic shock was significantly higher in patients with HAP compared to those without pneumonia (77.6% vs. 62%; *p* < 0.001). The rate of multisite colonization by CRAB at the time of admission was also significantly higher in patients with HAP (51% vs. 28.3%; *p* < 0.001). Additionally, the mean time from colonization to death was significantly shorter in HAP patients (13.5 ± 18.9 days) compared to those without pneumonia (17.2 ± 26.1 days; *p* < 0.001) and 20.5% of pneumonia patients were on NIV.

**Table 1:** Univariate analysis about patients with pneumonia compared to patients without pneumonia at 30 days

Variables	Pneumonia *n* = 392 (%)	No pneumonia *n* = 368 (%)	*p*
Age (mean), years	62.9 (± 15.7)	59.2 (± 17.8)	**0.002**
Male sex	260 (71.7)	264 (66.3)	0.117
Transfer in ICU from other wards	24 (85.7)	40 (66.7)	0.075
Previous hospitalization (90 previous days)	120 (30.6)	80 (21.7)	**0.006**
Previous ICU admission (90 previous days)	60 (15.3)	40 (10.9)	0.085
Previous surgery (30 previous days)	120 (31.9)	108 (30.7)	0.749
> 2 Comorbidities	205 (52.3)	204 (55.4)	0.586
Charlson Comorbidity Index (mean), points	8.3 (± 3.2)	3.4 (± 3.9)	**< 0.001**
SAPS index (mean), points	45.5 (± 13.9)	45.7 (± 15.1)	0.885
SOFA score (mean), points	7.3 (± 3.3)	6.3 (± 2.8)	**0.001**
QSOFA score (mean), points	1.9 (± 0.9)	2 (± 0.9)	**0.088**
Cardiovascular disease	184 (46.9)	164 (44.6)	0.513
Heart failure	84 (21.4)	44 (12)	**< 0.001**
Diabetes	104 (26.5)	80 (21.7)	0.128
Chronic kidney disease and dialysis	60 (15.3)	60 (16.3)	0.765
Cirrhosis	16 (4.1)	28 (7.6)	**0.043**
Neurological disease	16 (40)	20 (12.8)	**< 0.001**
Vasculitis	4 (5.3)	0 (0)	**0.007**
COPD	128 (32.7)	72 (19.6)	**< 0.001**
COVID-19	137 (34.9)	54 (14.6)	**< 0.001**
Neoplasm	48 (12.2)	64 (17.4)	0.052
Immunosuppressive therapy	216 (55.1)	136 (37)	**< 0.001**
Previous *Acinetobacter baumannii* colonization or infection (365 previous days)	8 (10.5)	16 (8.7)	0.642
Previous transplant (solid or hematologic)	0 (0)	20 (10.9)	**< 0.001**
Previous antibiotic therapy (30 days)	232 (59.2)	160 (43.5)	**< 0.001**
Fever > 37.5 °C	16 (40)	104 (66.7)	**0.003**
Sepsis	60 (16.9)	92 (27.1)	**< 0.001**
Septic Shock	304 (77.6)	228 (62)	**< 0.001**
Ventilatory support	354 (90.3)	232 (63)	**< 0.001**
CRRT	112 (32.7)	82 (27.3)	0.144
ECMO	32 (8.2)	28 (7.6)	0.790
*A. baumannii* multiple sites colonization	200 (51)	104 (28.3)	**< 0.001**
Time between colonization and death (mean), days	13.5 (± 18.9)	17.2 (± 26.1)	**0.006**
Length of hospitalization (mean), days	32.6 (± 22.2)	43.5 (± 37.7)	**< 0.001**
Length of ICU stay (mean), days	25.6 (± 21.2)	32.4 (± 32.2)	**< 0.001**
30-day mortality	278 (70.9)	219 (59.5)	**< 0.001**
In-hospital mortality	312 (79.6)	268 (72.8)	**0.033**

ICU: intensive care unit. COPD: chronic obstructive pulmonary disease. CRRT: continuous renal replacement therapies. ECMO: ExtraCorporeal Membrane Oxygenation

In Table 2, logistic regression analysis identified risk factors independently associated with HAP development. Increasing age was an independent risk factor (OR 1.018, CI 95% 1.007–1.029; *p* = 0.001); immunosuppressive therapy was also associated with a significantly increased risk of HAP (OR 2.287, CI 95% 1.618–3.231; *p* < 0.001), as was the need for ventilatory support (OR 3.191, CI 95% 1.883–5.407; *p* < 0.001). Finally, multisite CRAB colonization (OR 2.761, CI 95% 1.935–3.940; *p* < 0.001) emerged as a strong predictor of HAP development. Additionally, the presence of chronic obstructive pulmonary disease (COPD) (OR 1.755, CI 95% 1.177–2.616; *p* = 0.006) was associated with an increased risk of pneumonia.

**Table 2:** Logistic regression analysis about factors associated with pneumonia development and score points

Variables	OR	CI 95% Lower	CI 95% Upper	*p*	Score points
Age	1.018	1.007	1.029	0.001	1
Immunosuppressive therapy	2.287	1.618	3.231	< 0.001	2
Ventilatory support	3.191	1.883	5.407	< 0.001	3
Multisite colonization	2.761	1.935	3.940	< 0.0001	3
COPD	1.755	1.177	2.616	0.006	1

OR: odds ratio. CI: confidence interval. COPD: chronic obstructive pulmonary disease

**Fig. 1:**
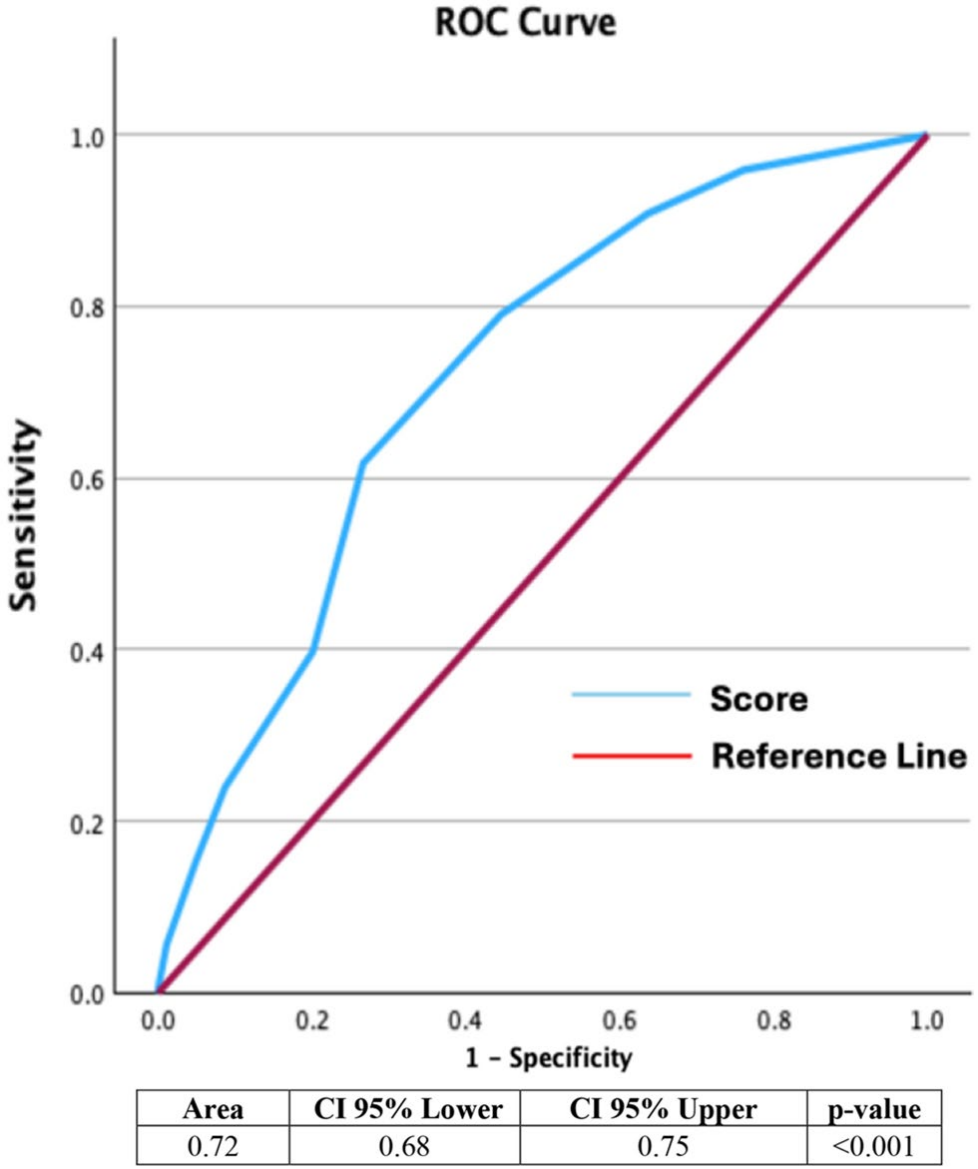
ROC curve of developed score. CI: confidence interval

The predictive score developed from the multivariate model was evaluated using the ROC curve, shown in Fig. 1. The area under the ROC curve (AUC) was 0.72 (95% CI 0.68–0.75; *p* < 0.001), indicating that the scoring system had moderate discriminatory power in predicting the development of HAP in CRAB-colonized patients, with a sensitivity of 79% and specificity of 55% with a score > 2. The maximum score was 10 points. The score was based on ß-coefficients of each variable obtained from the logistic regression analysis.

Table 3 compares patients with multisite CRAB colonization to those with colonization limited to the respiratory tract. Of the 760 patients, 304 (40%) had multisite colonization. Compared to patients with single-site colonization, those with multisite colonization had a significantly shorter mean ICU stay (25.5 ± 20.7 vs. 31.1 ± 30.6 days; *p* < 0.001) and a much higher incidence of septic shock (85.5% vs. 59.6%; *p* < 0.001). Patients with multisite colonization also had a significantly shorter mean time from colonization to death (12.2 ± 18.3 vs. 17.43 ± 25 days; *p* < 0.001), and the duration of antibiotic therapy was slightly longer in patients with multisite colonization (10.2 vs. 10.08 days), though this difference was not statistically significant (*p* = 0.685)

**Table 3:** Comparison between patients with multisite colonization compared to patients with only respiratory tract colonization

Variables	Multisite colonization *n* = 304 (%)	No multisite colonization *n* = 456 (%)	*p*
Age (mean), years	60.8 (± 12.7)	60.2 (± 13.4)	0.859
Male, sex	192 (63.2)	332 (72.8)	**0.005**
Transfer in ICU from other wards	8 (66.7)	56 (73.7)	0.729
Previous hospitalization (90 previous days)	88 (28.9)	112 (24.6)	0.180
Previous ICU admission (90 previous days)	56 (18.4)	44 (9.6)	**< 0.001**
Previous surgery (30 previous days)	132 (44)	96 (22.4)	**< 0.001**
> 2 Comorbidities	92 (30.2)	317 (69.5)	**< 0.001**
Charlson Comorbidity Index (mean), points	6 (± 4.5)	4 (± 4.1)	0.117
SAPS index (mean), points	46 (± 13.7)	45.3 (± 15.6)	0.515
SOFA score (mean), points	7 (± 3.0)	6.9 (± 3.3)	0.952
qSOFA score (mean), points	1.9 (± 1)	2.046 (± 0.9)	0.162
Cardiovascular disease	140 (46.1)	208 (45.6)	0.941
Heart failure	40 (13.2)	88 (19.3)	**0.029**
Diabetes	76 (25)	108 (23.7)	0.730
Chronic kidney disease and dialysis	28 (9.2)	92 (20.2)	**< 0.001**
Cirrhosis	12 (3.9)	32 (7)	0.082
Neurological disease	0 (0)	36 (20)	**0.047**
Vasculitis	0 (0)	4 (1.9)	1
COPD	64 (21.1)	136 (29.8)	**0.007**
Neoplasm	44 (14.5)	68 (14.9)	0.917
COVID-19	159 (52.3)	32 (7)	**< 0.001**
Immunosuppressive therapy	164 (53.9)	188 (41.2)	**< 0.001**
Previous *Acinetobacter baumannii* colonization or infection (365 previous days)	4 (9.1)	20 (9.3)	1
Previous transplant (solid or hematologic)	4 (9.1)	16 (7.4)	0.756
Previous antibiotic therapy (30 days)	152 (50)	240 (52.6)	0.505
Fever > 37.5 °C	8 (50)	112 (62.2)	0.423
Sepsis	32 (11.6)	120 (28.6)	**< 0.001**
Septic shock	260 (85.5)	272 (59.6)	**< 0.001**
Ventilatory support	244 (80.2)	342 (75)	0.053
CRRT	100 (38.2)	94 (24.7)	**< 0.001**
ECMO	28 (9.2)	32 (7)	0.275
Time between colonization and death (mean), days	12.2 (± 18.3)	17.43 (± 25)	**0.002**
Length of hospitalization (mean), days	32.9 (± 21.3)	41.2 (± 35.8)	**< 0.001**
Length of ICU stay (mean), days	25.5 (± 20.7)	31.1 (± 30.6)	**0.006**
30-day mortality	220 (72.3)	277 (60.7)	**< 0.001**
In-hospital mortality	252 (82.9)	328 (71.9)	**0.005**

ICU: intensive care unit. COPD: chronic obstructive pulmonary disease. CRRT: continuous renal replacement therapies. ECMO: ExtraCorporeal Membrane Oxygenation

**Fig. 2:**
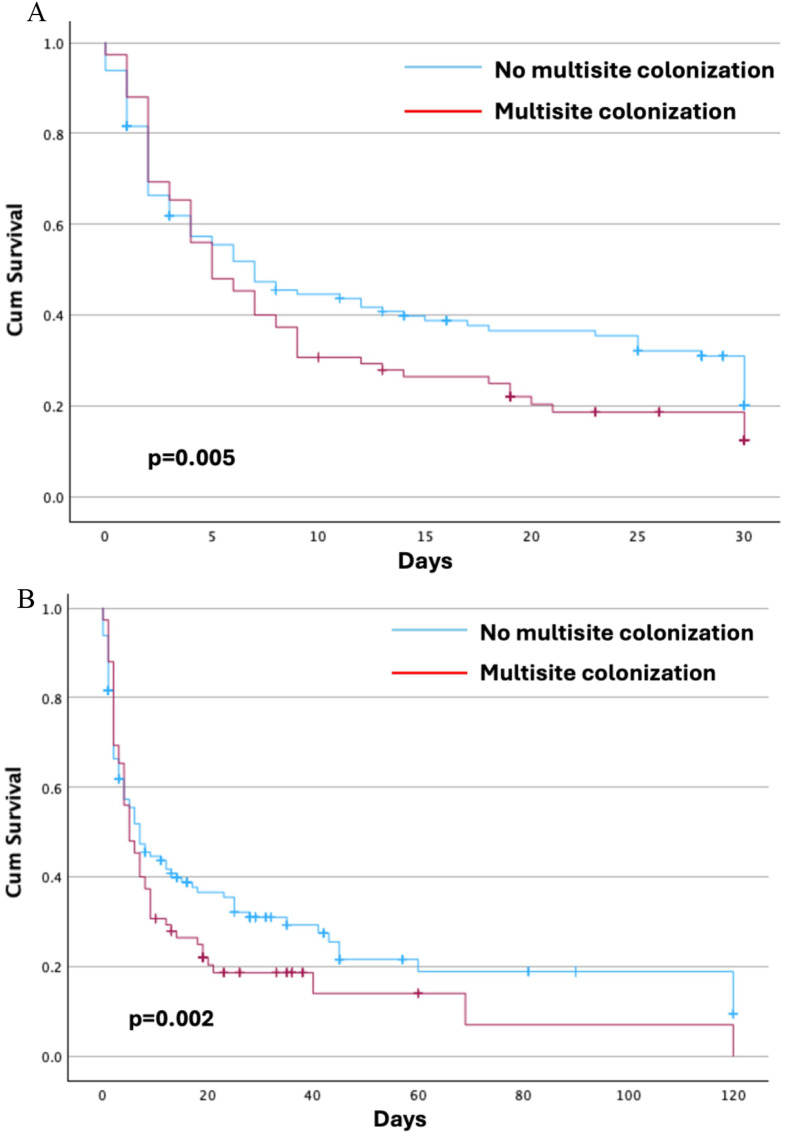
Kaplan-Meier curves* about survival in patients with or without multisite colonization at 30 days (**A**) and during hospitalization (**B**). *adjusted for sex, age, Charlson Comorbidity Index, COPD, COVID-19, immunosuppressive therapy, SOFA score, septic shock, length of ICU and hospitaly stay, antimicrobial therapies

Finally, Table 4 show multivariate analysis about risk factors associated with 30-day mortality. Kaplan-Meier survival curves (Fig. 2**)** highlight a significantly reduced survival in patients with multisite colonization compared to those with single-site colonization. At 30 days, patients with multisite colonization had worse survival outcomes (*p* = 0.05), and overall survival during hospitalization was also significantly lower (*p* = 0.02).

**Table 4:** Multivariate analysis about factors associated with 30-day mortality

VARIABLES	HR	CI 95% Lower	CI 95% Upper	*p*
Charlson Comorbity Index > 3	2.21	1.881	3.14	0.001
Transfer in ICU from other wards	2.27	1.684	3.32	< 0.001
Immunosuppressive therapy	2.122	1.843	4.427	0.01
Multisite colonization	3.461	2.935	4.95	< 0.0001
Septic shock	2.756	2.147	5.615	0.004

HR: hazard ratio. CI: confidence interval. ICU: intensive care unit

## Discussion

The results of this multicenter study offer valuable insights into the risk factors associated with the development of HAP in patients colonized by CRAB in ICU patients. This investigation highlights the substantial burden posed by CRAB colonization, particularly in critically ill patients, and underscores the clinical challenges of distinguishing between colonization and infection. Few studies in the literature explored differences between infection and colonization by CRAB, correlating with clinical outcomes [1, 8–11].

Our findings confirm that pneumonia in CRAB-colonized patients is common, affecting over half of the study population (56%), and is associated with significant in-hospital mortality, with rates approaching 80%. These results are consistent with previous studies, which have reported mortality rates between over 40% in patients with VAP caused by CRAB [12–18]. However, this study adds to the literature by identifying several independent risk factors for pneumonia development, including advanced age, immunosuppressive therapy, ventilatory support, and multisite colonization. These findings are important as they allow clinicians to stratify the risk of pneumonia in colonized patients and consider early preventive strategies or more aggressive therapeutic interventions.

Age emerged as a significant predictor of pneumonia development. This finding is expected, as older patients often have a reduced capacity to mount an effective immune response against bacterial pathogens, particularly in the context of severe infections like those caused by CRAB [19]. Moreover, immunosuppressive therapy also stood out as a major risk factor. The role of immunosuppression in CRAB infections cannot be overlooked. Immunosuppressive therapies, commonly used in patients with autoimmune diseases, malignancies, or after organ transplantation, impair both innate and adaptive immune responses. This significantly compromises the host’s ability to clear bacterial colonization and promotes the transition from colonization to invasive infection [20]. The decline of innate lung immunity with age is driven by “inflamm-aging,” a state of chronic, low-grade inflammation. This impairs microbial recognition, response, and clearance. Age-related changes in immune cells, like antigen-presenting cells and neutrophils, further weaken defenses. The lung environment itself changes, with reduced mucociliary clearance hindering pathogen removal. Changes in complement, surfactant proteins, and the lung microbiome also contribute to decreased immunocompetence [21]. Our findings highlight the necessity of close monitoring and potentially earlier interventions in this population to mitigate the risk of HAP development [22].

Ventilatory support, particularly mechanical ventilation, was another independent predictor of pneumonia. CRAB is known to thrive in hospital environments, particularly on ventilator equipment, where it can persist and be transmitted to patients [23]. The need for ventilatory support also indicates the severity of the underlying disease, which may contribute to the risk of infection. Additionally, prolonged mechanical ventilation disrupts the normal defense mechanisms of the respiratory tract, including mucociliary clearance, and allows CRAB to colonize and infect the lower respiratory tract more easily [24]. Our findings also show that patients on NIV were at a lower but still significant risk of pneumonia (20.5% of pneumonia patients were on NIV). This suggests that, while NIV may be less invasive, it still poses a risk for respiratory tract colonization and subsequent infection. These results underscore the importance of stringent infection control measures in patients requiring any form of respiratory support to prevent the transition from colonization to infection [25].

One of the most important findings of our study is the association between multisite colonization and the development of pneumonia. This adds to the growing body of evidence suggesting that multisite colonization by CRAB significantly increases the risk of subsequent infection. The colonization of multiple anatomical sites, such as the respiratory tract, skin, and gastrointestinal tract, may reflect a higher bacterial load and a greater likelihood of bacterial translocation across mucosal barriers [26]. Furthermore, multisite colonization could also indicate more widespread environmental contamination, increasing the risk of cross-contamination between colonized sites [27]. Our subgroup analysis showed that patients with multisite colonization had worse outcomes, including higher ICU admission rates and a shorter time from diagnosis to death compared to those with single-site colonization. This suggests that multisite colonization is not only a marker of disease severity but also a predictor of poor prognosis. Interestingly, the presence of multisite colonization may also reflect more complex underlying pathophysiological processes, including impaired host defenses and prolonged exposure to invasive procedures or broad-spectrum antibiotics. These findings point to the need for early identification of CRAB colonization at multiple sites. Multisite colonization could serve as an early warning sign for clinicians, prompting closer surveillance and more aggressive measures to prevent the progression to pneumonia. Future studies could focus on interventions targeting patients with multisite colonization to reduce the risk of infection, also BSI, such as enhanced decolonization protocols or more frequent microbiological screening during their ICU stay [28].

The clinical implications of our study are significant. Identifying high-risk patients using the risk factors described in our multivariate model can help guide clinical decision-making. For instance, patients with a high-risk profile may benefit from intensified preventive strategies, including more frequent microbial surveillance, the early initiation of empiric antibiotic therapy, or enhanced infection control measures. Additionally, our study provides a scoring system based on these risk factors that could be used to predict pneumonia development in CRAB-colonized patients. The area under the curve (AUC) of 0.72 suggests that this scoring system has reasonable predictive accuracy, although further validation in larger, prospective studies is warranted. Incorporating such a score into routine clinical practice could help stratify patients according to their risk of developing pneumonia and inform more personalized treatment plans.

There is also a clear need for future research to explore the role of antimicrobial stewardship in reducing the risk of pneumonia in CRAB-colonized patients. Given the high rates of antibiotic resistance in CRAB, selecting appropriate antibiotic regimens remains challenging. Studies focusing on the use of combination therapies or novel antimicrobials, such as cefiderocol, in high-risk populations could provide valuable insights into improving patient outcomes [29, 30].

While our study provides important data, several limitations should be acknowledged. First, the retrospective nature of the study introduces the possibility of selection bias (i.e. duration of ventilation support). Second, the study was conducted in Italian hospitals, which may limit the generalizability of the findings to other settings with different CRAB epidemiology or healthcare practices. Third, microbiological techniques for detecting CRAB colonization varied slightly between centers and have changed over time, which could have led to differences in the sensitivity and specificity of colonization detection. Finally, there is a long time span that includes the pandemic period and the introduction of new therapies, which introduce a certain degree of variability in the study population. This should be considered as an important limitation.

Despite these limitations, this multicenter study identifies key risk factors for the development of hospital-acquired pneumonia in ICU patients colonized by CRAB. These findings underscore the importance of early identification and risk stratification in colonized patients to guide targeted interventions aimed at preventing the progression to pneumonia. Enhanced infection control measures, vigilant monitoring of colonized patients, and appropriate antibiotic stewardship are critical in mitigating the impact of CRAB in the ICU. Our study also introduces a scoring system that can help clinicians predict which patients are at greatest risk of developing pneumonia, providing a valuable tool for optimizing care in this vulnerable population. Further research should focus on refining this risk score and exploring novel therapeutic strategies for preventing and treating CRAB-related infections [31, 32].

## Data Availability

Data are available on request at a.russo@unicz.it.
